# Practical Points

**Published:** 1907-04-06

**Authors:** 


### PRACTICAL POINTS.

### THE COST OF AN OCCUPIED HOSPITAL BED, AND HOW TO CONTROL IT.

The chairman of a large provincial hospital writes: I have again, as for many years past, been carefully studying your last edition of "Hospitals and Charities," and find the hospital, of which I have been chairman for fifteen years, again stands high in the list for cost per bed occupied. It has not always been so. We have varied from £55 to £70, and this with the same people using the same care, and the difficulty is to know how or why these great variations. It is not from want of trouble on the part of the managers. It is, however, very annoying. I write to suggest that you would confer further benefit to those who are doing their best to run their hospitals efficiently and economically if you would get someone well up in all the details to undertake the examination of individual hospital accounts and the giving advice to the managers on their weak points and showing where saving might be effected. You have the detailed accounts, which we have not got, and could therefore say why, for instance, A and B can feed their patients for £15 per bed, while it costs C £25, etc. Kindly excuse my writing to you about this—my only excuse is the interest I take in the work.

*** The great variations referred to are due to different systems, and the presence or absence of a relatively complete or incomplete method of checking expenditure, based upon a knowledge of prices and how to buy. Owing to the action of King Edward's Hospital Fund in London, Economy Committees have been set up in connection with several London hospitals, with the best results. It might be useful for the writer to obtain a copy of "Hospital Expenditure: The Commissariat," published by the Scientific Press, 28 and 29 Southampton Street, Strand, and to study it carefully. It is full of practical information. In regard to the suggestion of an inquiry into the details of individual hospital accounts and advice to managers on their weak points, with a view to indicate where saving might be effected, we may point out that Sir Henry Burdett has undertaken this work with the aid of his staff, for a great many years, and is constantly rendering help in this way.—Ed. The Hospital.

